# A Novel Method for the Determination of the Lateral Dimensions of 2D Rectangular Flakes

**DOI:** 10.3390/ma15041560

**Published:** 2022-02-19

**Authors:** Thanasis D. Papathanasiou, Andreas Tsiantis, Yanwei Wang

**Affiliations:** 1 Department of Mechanical Engineering, University of Thessaly, 383-34 Volos, Greece; armitatz@gmail.com; 2 Department of Chemical and Materials Engineering, School of Engineering and Digital Sciences, Nazarbayev University, Nur-Sultan 010000, Kazakhstan; yanwei.wang@nu.edu.kz

**Keywords:** flake composites, composites characterization, microstructure, flake alignment, flake dimensions

## Abstract

We present a novel method for the determination of the lateral dimensions of thin rectangular flakes, as they exist randomly dispersed in flake composites. Knowledge of flake size and shape is essential for the correct prediction of the mechanical, electrical, thermal and barrier properties of flake composites. The required information is the distribution function of lengths of the lines representing the intersection of flakes with a sectioning plane, as seen in cross-sections of composite samples used in optical or electron microscopy or obtained using tomographic imaging techniques. The key observation is that the major peak of the distribution function coincides with the short dimension *S* of the flake while a secondary peak corresponds to its long dimension *W*. These observations are explained using Monte-Carlo simulations, as well as deterministic, geometry-based modeling and probability analysis. Since the strength of the secondary peak diminishes with increasing flake aspect ratio r=W/S, we develop two additional methods for the determination of *W*. The first finds *W* from the maximum intersection length; this procedure is justified by computing the relevant probability fields through Monte-Carlo simulations. The second method finds *r* from the average intersection length and is valid in the range 1<r<15. The performance of these techniques is tested and found to be very good using blind experiments in numerically sectioned specimens.

## 1. Introduction

The problem of obtaining the dimensions of filler particles in composite materials from information contained in two-dimensional cross-sections, typically obtained for and used in optical, confocal, scanning-electron and transmission microscopy or in tomographic imaging, is a long-standing problem in composites science. The reason for this is self-evident, since knowledge of the size, shape and orientation of the reinforcing particles is a basic requirement for any reasonable estimation of the properties of the resulting composites. This problem has been handled in sufficient detail in the case of fibrous composites, in two-dimensional (2D) cross-sections of which the fibers appear as circles or ellipses, depending on their orientation with respect to the sectioning plane [1,2,3,4,5,6,7]. In contrast, this problem has not been addressed at all in the case of composites in which the reinforcing particles have a thin, planar shape (flake composites), even though these materials find significant applications in diverse fields (packaging films and containers, fire resistant materials, anti-corrosion paints, as anti-oxidants and UV-protection agents in polymers etc.) [8,9,10]. For example, use of metallic flakes in conductive composites and electromagnetic interference (EMI) shielding applications, hinges on correct estimation on the in-situ lateral dimensions of the flakes, as they directly impact the percolation threshold of the material [11,12,13,14]. Use of flake composites as barrier materials relies on their large surface area-to-volume ratio, which increases dramatically the tortuosity of the medium and the length of the diffusion path of the penetrant molecules, with a corresponding decrease in the effective diffusion coefficient. We have recently demonstrated [15,16] that, in the particular case of flakes of rectangular shape, the barrier efficiency of the resulting composite is a very strong function of the planar flake aspect ratio. Specifically, we have shown that, when the Barrier Improvement Factor (BIF $∼1/D_{\mathrm{eff}}$), where $D_{\mathrm{eff}}$ is the effective diffusion coefficient of the penetrant molecules in the composite, is expressed as a quadratic polynomial of (αϕ), namely $\mathrm{BIF}=1+C_{1}\alpha \phi +C_{2}{\left(\alpha \phi \right)}^{2}$, where α is an aspect ratio based on the thickness of flake, and ϕ is the flake volume fraction. The coefficients $C_{1}$ and $C_{2}$ depend on the lateral aspect ratio *r*, namely, $C_{1}\;∼1/r$ and $C_{2}\;∼1/r^{2}$. The inverse quadratic dependence of $C_{2}$ on *r* implies that the effectiveness of rectangular flakes as barrier materials diminishes rapidly as their aspect ratio increases. Information about the lateral dimensions of flakes is only rarely available in processed composites, even though substantial information exists regarding the shape of as-synthesized flakes, especially those produced by liquid phase exfoliation. Meunier et al. [17] have demonstrated that the lateral dimensions of graphene nanoflakes can be controlled in a thermal plasma reactor. Lin et al. [18] have presented detailed characterization of graphene flakes using microscopy (TEM and optical) as well as AFM and Raman micro-spectroscopy; the lateral dimensions, in terms of the Feret diameter, were shown to be in the range of 1 μm. Jiang et al. [19] have presented pictures of SnSe rectangular flakes with lateral dimensions 50 μm× 30 μm and thickness at the atomic level. Zeng et al [20] have shown pictures of polygonal MoS2 sheets with lateral dimensions ∼ 150 μm in aqueous solution. Peng et al. [21] have shown TEM images of polygonal flakes of 2D Titanium Carbide with lateral dimensions 1–6 μm. Mag-Isa et al. [22] have presented images of as-produced graphene and MoS${}_{2}$ flakes with lateral dimensions around 100 and 50 μm, respectively. Length distributions were also reported. The need to properly quantify the shape of flake-like 2D materials in a statistically meaningful sense and beyond the use of the Feret diameter has been addressed by Santos et al. [23] and has been identified as a strong prerequisite for the standardization of feedstock in the evolving 2D materials industry. In the case of flakes as they exist in processed composites or coatings, the in-situ size and shape of the flakes can be different of those of the starting material, due to attrition.

**Figure 1:**
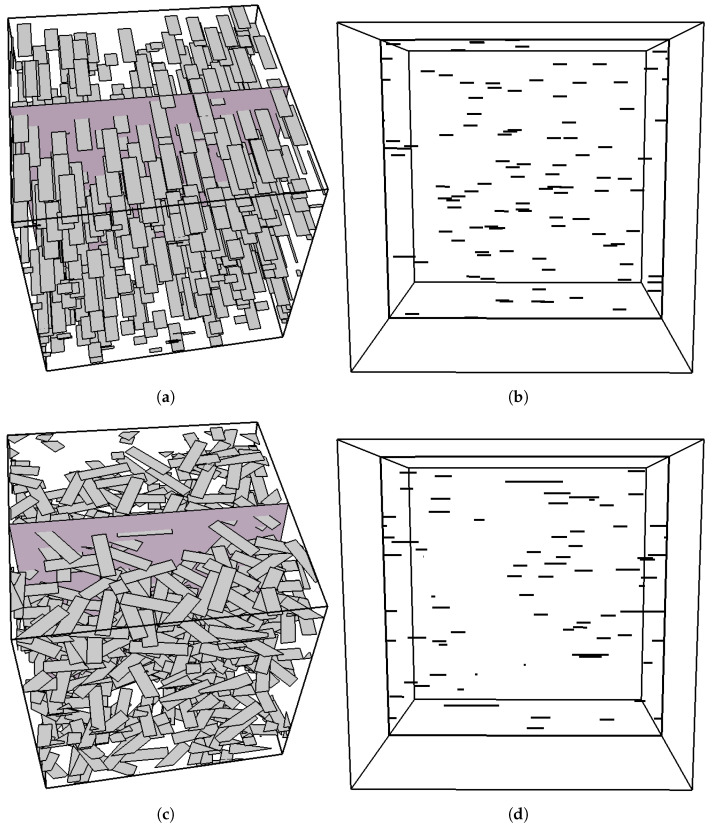
Typical 3D RVEs including flakes oriented parallel to the *X*-*Z* plane, showing the sectioning plane and the resulting intersections. From left to right: (**a**) A flake composite with unidirectional flakes of aspect ratio r=4, (**b**) The intersecting plane shows a collection of parallel lines of equal length, (**c**) a similar composite in which the flakes assume random in-plane orientations, and (**d**) the corresponding image of the intersecting plane where the intersections now appear as parallel lines of a variety of lengths. In this illustration, the RVE contains 500 flakes and there are ∼40 intersections.

Flakes in composite parts are typically imaged by sectioning the composite and observing either a fracture surface or a polished cross section using optical, or scanning/transmission electron microscopy [24,25,26,27]. In such cross-sections, 2D rectangular flakes will appear as lines, as shown schematically in Figure 1. While this approach can yield the flake dimensions for perfectly aligned flakes, as in this case the intersections of the flakes with a perpendicular sectioning plane will appear as lines of equal length (Figure 1a,b), this procedure clearly breaks down when the flake orientations deviate from perfect alignment. This is shown in Figure 1c,d, in which it is clear that when the flakes assume random in-plane orientations, the cross-section consists of line segments having a distribution of lengths. It is currently not possible to back-calculate the lateral dimensions of flakes from images of cross sections such as shown in Figure 1d.

The purpose of this paper is to overcome this shortcoming and to present the development and testing of a procedure which allows the extraction of these parameters (*S*, *r*), from the statistics of the length distribution seen in cross-sections of composites (e.g., Figure 1). The assumptions underlying this work are:

(i) Flakes are of rectangular shape and of uniform size;
(ii) Flakes are parallel to each other;
(iii) Flakes have random in-plane orientations in the interval [−π/2,+π/2].

In the following sections we first present results of numerical sectioning experiments in realistic three-dimensional (3D) Representative Volume Elements (RVEs), in which the statistical features of the distribution of the intersection lengths are revealed. Following this, we develop geometry-based models for the statistics of the intersection lengths; these, implemented in a Monte-Carlo environment, shed light into the mechanism(s) causing the characteristic distributions seen in computationally sectioned samples. Using these results we develop and test methodologies for determining the flake aspect ratio from the maximum intersection length, and also from the average intersection length.

## 2. Geometry Generation and Numerical Sectioning

**Figure 2:**
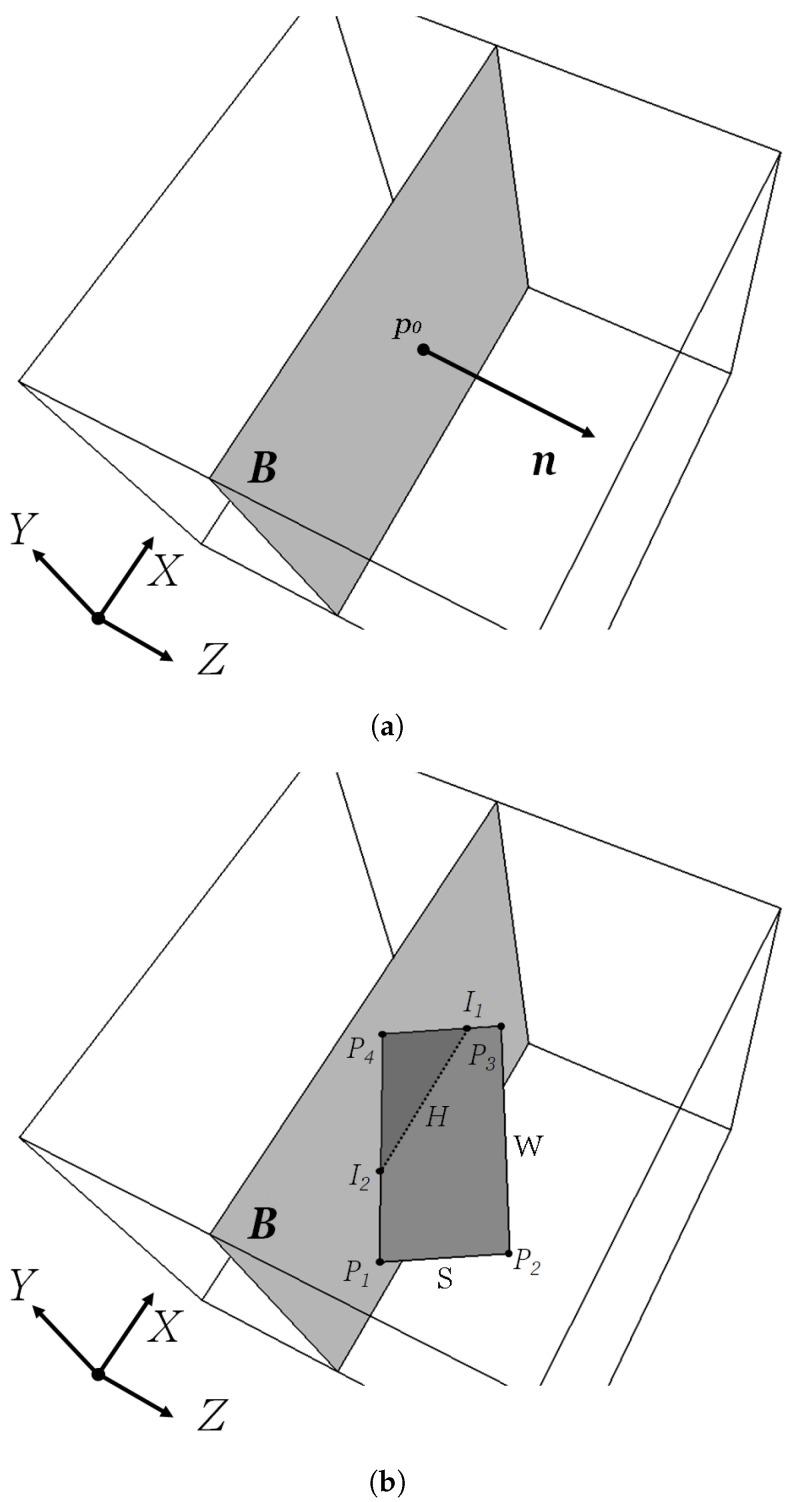
Schematic illustrating the calculation of the intersection length (*H*) in a sectioning experiment. (**a**) A unit cell with the sectioning plane (**B**). The unit vector **n** located at point $p_{0}$ is shown. (**b**) Intersection of plane (**B**) with a flake, defined by the four points $P_{1}$–$P_{4}$ and having a short side (*S*), long side (*W*) and aspect ratio r=W/S. The flake intersects plane (**B**) at points $I_{1}$ and $I_{2}$.

3D RVEs are generated using an in-house Random Sequential Addition (RSA) algorithm. The RSA process is implemented as described in a previous work [28] with the flakes allowed to assume random in-plane orientations. A fail-safe mechanism is implemented by which the calculation stops if a specified number of attempts (O($10^{9}$)) is exceeded. For numerical sectioning, as shown in the schematic in Figure 2, a plane (**B**) normal to the plane of the flakes is placed at some location within the RVE; plane **B** is described in vector form as the set of points *p* for which $(p-p_{0})\;\cdot \;n=0$ where **n** is the unit normal vector and $p_{0}$ is a point on the plane. The distance (*h*) between each flake center and the plane **B** is computed. For each flake satisfying h<D/2, where *D* is the length of the flake diagonal, the four line segments between the corner points of the flake ($\overset{↔}{P_{1}-P_{2}},\overset{↔}{P_{2}-P_{3}},\overset{↔}{P_{3}-P_{4}},\overset{↔}{P_{4}-P_{1}}$) that describe the flake boundaries are checked for possible intersection with plane **B**.

For each line segment forming the perimeter of the rectangle, the direction vector $\vec{d}=\vec{P_{k}P_{m}}$ is computed and each such line is described as the set of points *X* for which $X=P_{k}+t\;\cdot \;\vec{d},t\in R$. Line-plane intersections are determined by substitution of the line equation to the plane equation and solving for *t*, namely:

(1) $$(\left(P_{k}+t\;\cdot \;\vec{d}\right)-p_{0})\;\cdot \;n=0$$

(2) $$t=\frac{(p_{0}-P_{k})\;\cdot \;n}{\vec{d}\;\cdot \;n}$$

In the case where the denominator in Equation (2) is zero, then, if the numerator is also 0 the line is coplanar with the plane **B**, while if the numerator is not 0 the line is parallel to plane **B**. Both such cases are of no interest in this work. Obviously, if one flake boundary segment intersects plane **B** then there will be another flake segment that will also intersect plane **B**; therefore the intersection points, if they exist, will always appear in pairs ($I_{1}$ and $I_{2}$). The two intersection points ($I_{1},I_{2}$), with coordinates ${\left(x,y,z\right)}_{1}$ and ${\left(x,y,z\right)}_{2}$ respectively, are found from Equation (3) and are used to calculate the length *H* of the intersection segment (Figure 2).

(3) $$I_{n}=P_{k}+t\;\cdot \;\vec{d}$$

**Figure 3:**
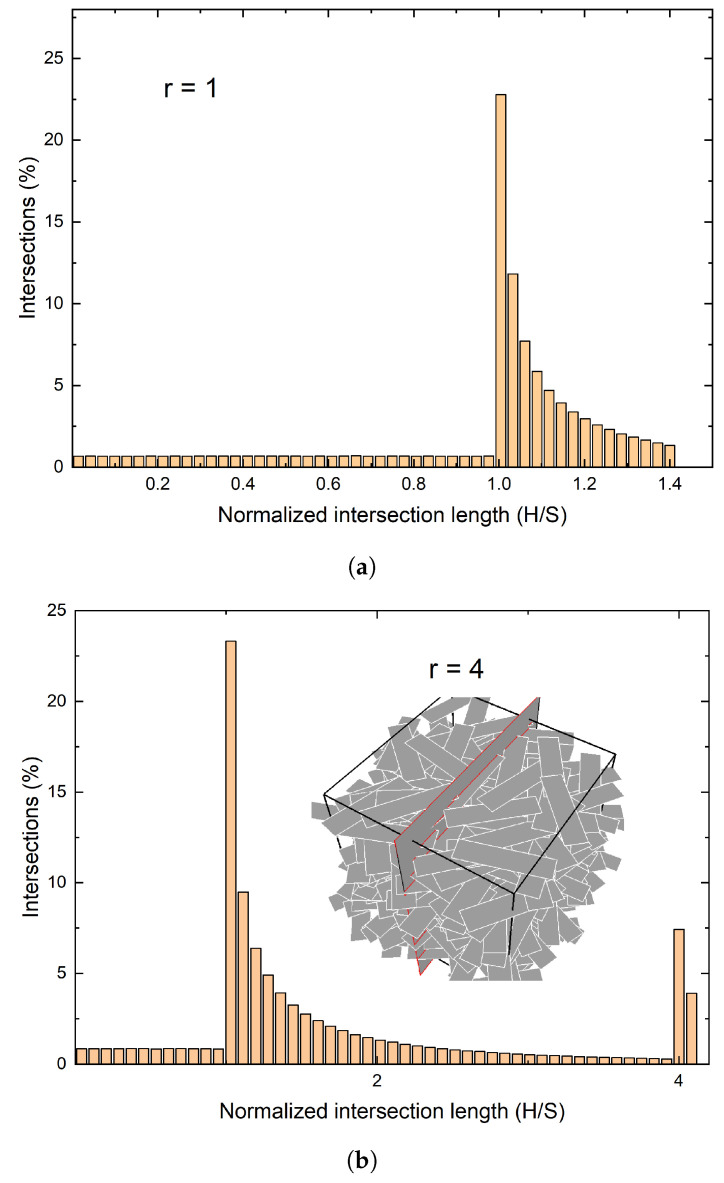

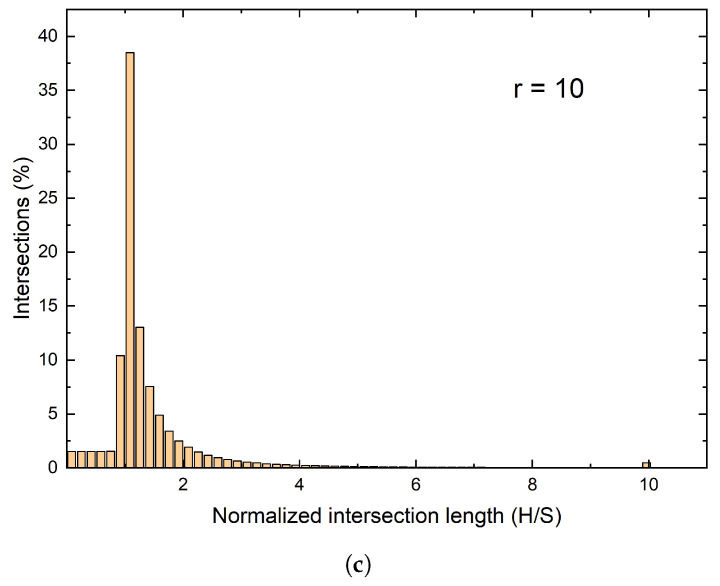
Distribution of intersection lengths for various flake aspect ratios (r=S/W). Each geometry contains 50,000 flakes. The intersection lengths (horizontal axis) have been normalized by dividing with width *S* of the flakes. (**a**) When r=1 the two peaks are merged resulting in a maximum at H=S. (**b**) for r>1 there is a second maximum at *r*. (**c**) when r>>1 the second maximum becomes smaller and eventually diminishes as *r* increases.

Typical distributions of the intersection lengths are shown in Figure 3, for three values of *r*. Each such histogram shows three distinct areas; the first lies to the left of the first peak, which invariably occurs at the point at which the intersection length *H* equals the flake width *S*. This corresponds to intersection segments of length H<S. The second area lies between the first and the second peak (which corresponds to H=W), as it can be easily seen in Figure 3a,b and less clearly in Figure 3c. The third area corresponds to H>W with *H* approaching the length of the flake diagonal-–this is the longest intersection length that might occur. In the limiting case of r=1 the two peaks at *S* and *W* coincide.

Figure 3 shows that the main peak in the histogram of the intersection lengths coincides with the small dimension of the flake (*S*), while the second peak coincides with the long dimension (*W*) of the flake. This result has been reproduced in thousands of simulations, in RVEs containing from $∼10^{3}$ to $∼10^{7}$ flakes and for flake aspect rations ranging from 1 to 40. As *r* increases, the second peak becomes smaller and eventually disappears. This appears to limit the usefulness of this observation to flakes of small (<5) aspect ratio. However, we will show that by correlating the maximum intersection length, max(H) and/or the average intersection length $H_{av}$ to the flake aspect ratio, we will offer a method for determining the long dimension of the flake at higher values of *r*. This will be discussed in following sections.

## 3. Theoretical Model for the Sectioning Process

**Figure 4:**
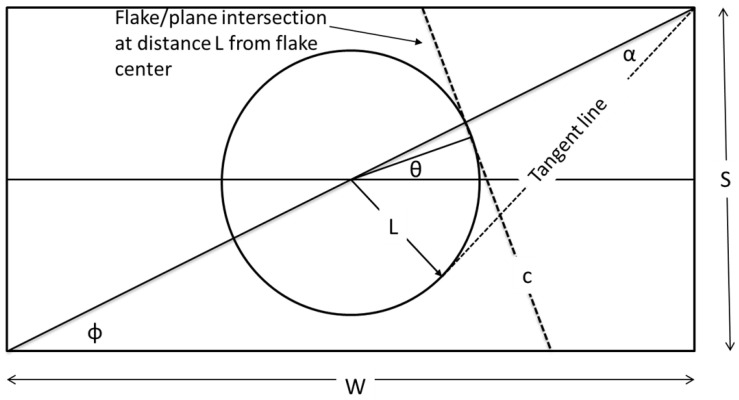
Basic geometrical features of the problem. The intersection between the cutting plane and the plane of the flake (line (c) in the schematic) will be tangent to a circle of radius *L*, centered at the center of the flake. The counter-clockwise angle formed between the long axis of the rectangle and line c is π/2+θ. Thus, θ=0 corresponds to line (c) being perpendicular to the long axis of the rectangle, while, when θ=π/2, line (c) is parallel to the long axis of the rectangle.

In the following we derive expressions for the length *H* of the line segment forming the intersection between a flake and plane **B**. While the problem of determining *H* can be handled with ease by computational geometry, as shown in the previous section, in this segment we will develop explicit expressions for *H* in terms of the orientation (θ) of the sectioning plane and its distance *L* from the center of the flake; both *L* and θ are random variables and thus, while deterministic in form, the relevant model will be subsequently evaluated in a Monte-Carlo context. In the process we will offer an explanation for the observations of Figure 3 and also, derive correlations between max(H) and/or $H_{av}$ and the flake aspect ratio. The basic geometrical features of the problem are shown in Figure 4. The angle between the diagonal and the long side of the rectangle is ϕ=atan(1/r). The intersection between the rectangle and the intersecting plane is indicated as line (c). If *L* is the distance from the center of the flake to this line, then line (c) will be tangent to a circle of radius *L* centered at the center of the rectangle. The angle formed between the diagonal of the rectangle and the tangent drawn from a corner of the rectangle to a circle of radius *L*, centered at the center of the rectangle, is α=asin(2L/D) where $D=S\sqrt{1+r^{2}}$ is the length of the diagonal of the flake.

**Figure 5:**
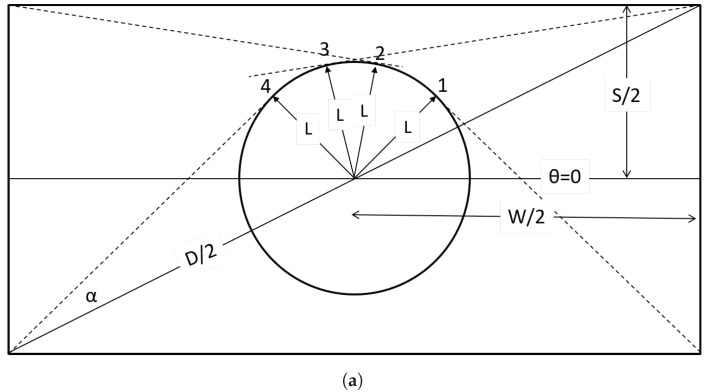

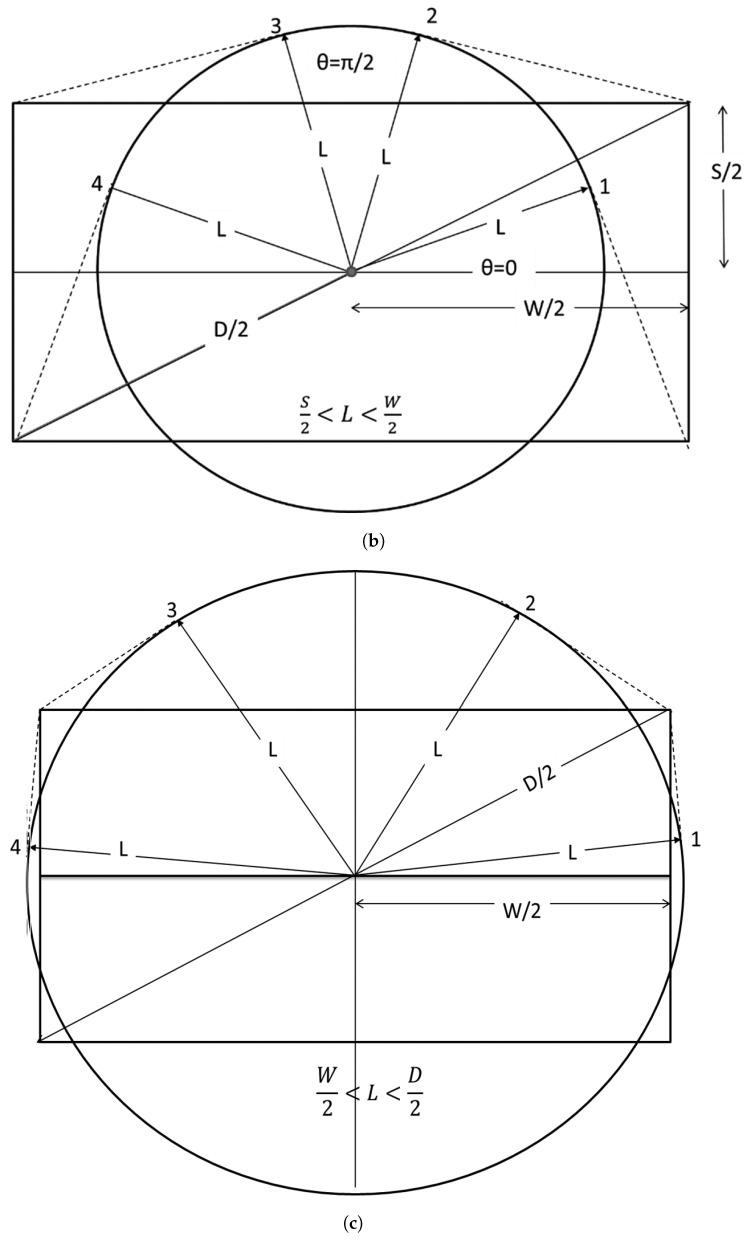
Schematic illustrating the steps outlined in (i) to (iii) above. Broken lines indicate the tangents to the circle of radius (*L*) originating from the corners of the rectangle, as shown. In cases (**b**,**c**), only planes with orientations corresponding to $\theta_{1}\;<\;\theta \;<\;\theta_{2}$ and $\theta_{3}\;<\;\theta \;<\;\theta_{4}$ will intersect the rectangle. The angle θ is counter-clockwise, formed between the long axis of the rectangle and the radius of the circle at θ, 0<θ<π.

Obviously, a necessary condition for intersection between line (c) and the rectangle is that L<D/2. This condition is necessary but not sufficient as the orientation of the cutting plane (the slope of line (c)) must also be suitable. Depending on the distance (*L*) from the center of the rectangle, not all possible angles (θ) will result in an intersection with the rectangle. With reference to Figure 5 we distinguish three regions:

(i) L<S/2 (Figure 5a).
  In this case it is obvious that all lines tangent to the circle with radius (*L*) will intersect the rectangle; therefore, for L<S/2 there will be an intersecting line for all θ, 0<θ<π. We define as angles $\theta_{1}$ to $\theta_{4}$ the angles formed (counter-clockwise) between the long axis of the rectangle and the arc points 1–4, at which a tangent will pass through a corner of the rectangle, as shown in Figure 5a. It can be shown that $\theta_{1}\;=\;\pi /2\;-\;\alpha \;-\;\phi$, $\theta_{2}\;=\;\pi \;-\;\phi \;-\;\mathrm{acos}\left(2L/D\right)$, $\theta_{3}\;=\;\pi /2\;-\;\alpha \;+\;\phi$, $\theta_{4}\;=\;\pi /2\;+\;\alpha \;+\;\phi$. It then follows that the intersection length can be calculated as:
  (a) $0\;<\;\theta \;<\;\theta_{1}$
    (4) $$H=\frac{S}{\cos(\theta )}$$
  (b) $\theta_{1}\;<\;\theta \;<\;\theta_{2}$
    (5) $$H=\left(D-\frac{2L}{\cos(\phi -\theta )}\right)\frac{\cos(\phi -\theta )}{\sin(2\theta )}$$
  (c) $\theta_{2}\;<\;\theta \;<\;\theta_{3}$
    (6) $$H=\frac{W}{\sin(\theta )}$$
  (d) $\theta_{3}\;<\;\theta \;<\;\theta_{4}$
    (7) $$H=\left(D+\frac{2L}{\cos(\phi +\theta )}\right)\frac{\cos(\phi +\theta )}{\sin(2\theta )}$$
  (e) $\theta_{4}\;<\;\theta \;<\;\pi$
    (8) $$H=\frac{-S}{\cos(\theta )}$$
  Equations (4), (6) and (8) represent the case when the cutting plane intersects two opposite sides of the rectangle, while Equations (5) and (7) represent the case when it intersects adjacent sides. Only Equations (5)–(7) are expected to produce long intersections, while Equations (4) and (8) might generate intersections with lengths comparable to *S*.
(ii) S/2<L<W/2 (Figure 5b).
  In this case, not all tangent lines to the circle of radius (*L*) will intersect the rectangle. From Figure 5b it is clear that no intersection will occur if $\theta_{2}\;<\;\theta \;<\;\theta_{3}$. For all other values of θ the cutting plane will intersect the rectangle. It can be shown that $\theta_{1}\;=\;\pi /2\;-\;\alpha \;-\;\phi$, $\theta_{2}\;=\;\phi \;+\;\mathrm{acos}\left(2L/D\right)$, $\theta_{3}\;=\;\pi \;-\;\theta_{2}$, $\theta_{4}\;=\;\pi /2\;+\;\alpha \;+\;\phi$. In this case the intersection lengths are calculated as:
  (a) for $0\;<\;\theta \;<\;\theta_{1}$ use Equation (4);
  (b) for $\theta_{1}\;<\;\theta \;<\;\theta_{2}$ use Equation (5);
  (c) for $\theta_{3}\;<\;\theta \;<\;\theta_{4}$ use Equation (7);
  (d) for $\theta_{4}\;<\;\theta \;<\;\pi$ use Equation (8);
  (e) for all other angles H=0.
(iii) W/2<L<D/2 (Figure 5c).
  The situation is similar to (ii) and the arcs at which no intersecting lines can be drawn are $\theta_{2}\;<\;\theta \;<\;\theta_{3}$, $0\;<\;\theta \;<\;\theta_{1}$, and $\theta_{4}\;<\;\theta \;<\;\pi$. $\theta_{1}\;=\;\pi /2\;-\;\alpha \;-\;\phi$, $\theta_{2}\;=\;\phi \;+\;\mathrm{acos}\left(2L/D\right)$, $\theta_{3}\;=\;\pi \;-\;\theta_{2}$, $\theta_{4}\;=\;\pi /2\;+\;\alpha \;+\;\phi$. The intersection lengths are
  (a) for $\theta_{1}\;<\;\theta \;<\;\theta_{2}$ use Equation (5);
  (b) for $\theta_{3}\;<\;\theta \;<\;\theta_{4}$ use Equation (7);
  (c) for all other angles H=0.

**Figure 6:**
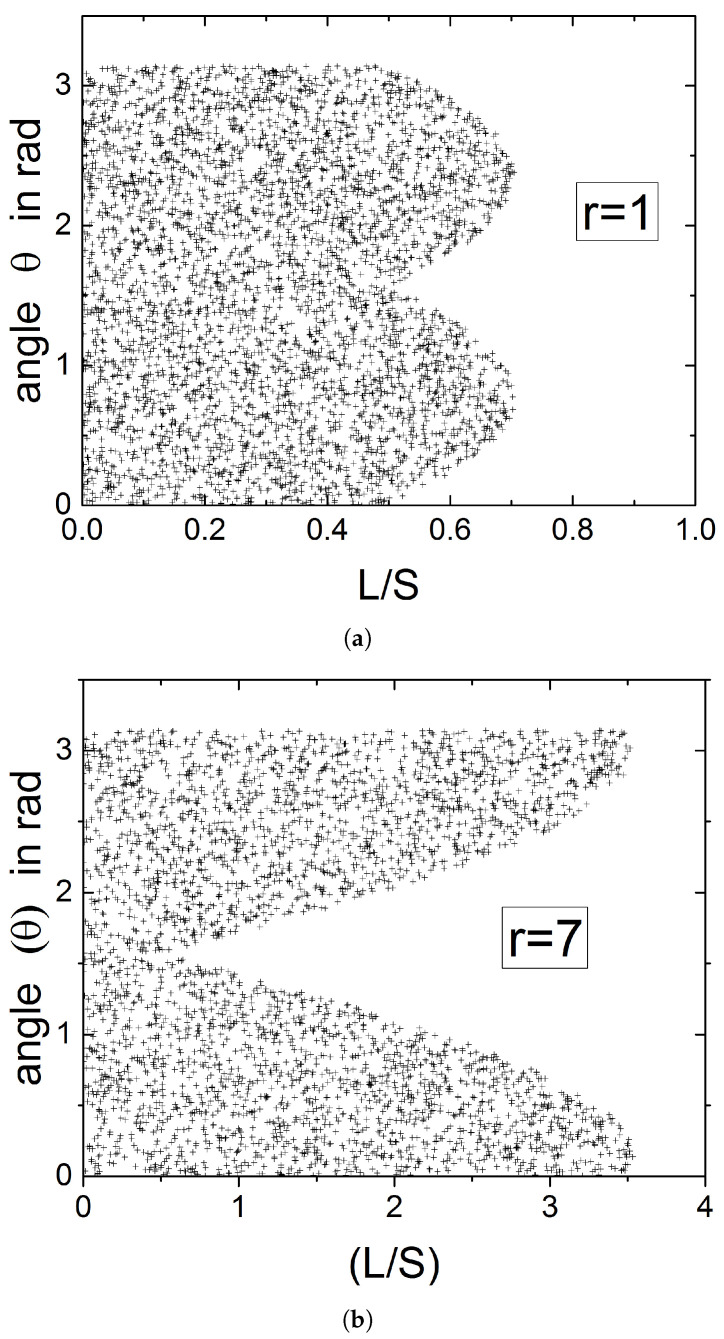
Correlation between the (normalized) distance of the center of the flake from the intersecting line (L/S, horizontal axis) and the orientation of the flake (θ in rad, vertical axis), for situations that have resulted in intersection. Each point in the graphs represents one intersection between the flake and the cutting plane. Shown are results for a total of *N* = 5000 random combinations of (*L*, 0<L<D/2) (**a**) and (θ,0<θ<π) (**b**) that have resulted in ∼4400 intersections.

From the above it is evident that in reproducing the sectioning experiment that has led to the histograms of Figure 3, the distance *L* and the orientation θ of the intersecting line are not independent. This is demonstrated in Figure 6 in which the angle θ is plotted against the distance *L*, for all cases that have resulted in an intersection. It is seen that at distances L<S/2, all angles between the cutting plane and the flake will result in an intersection. This range shrinks as the distance of the flake center from the cutting plane increases, and at L>W/2, only a very narrow set of θ will yield intersections.

## 4. Results and Discussion

### 4.1. General Observations

**Figure 7:**
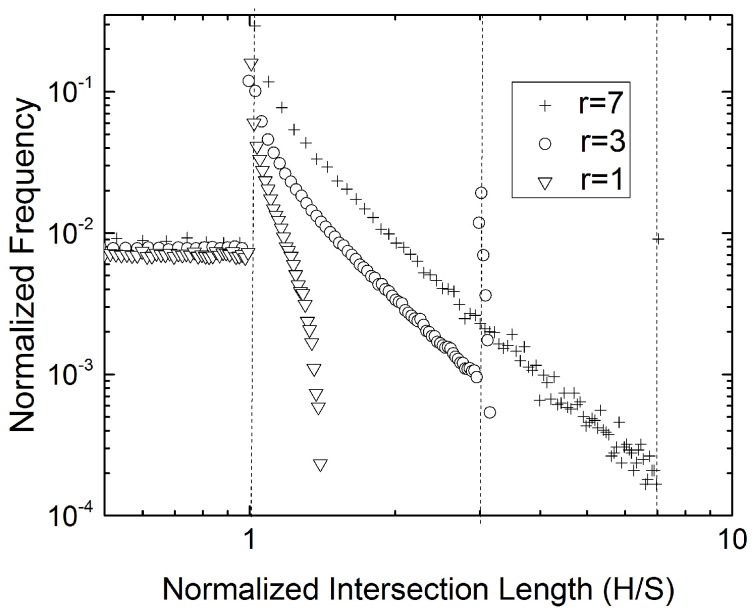
Frequency of intersection lengths for three cases, r=1, r=3, and r=7. $N=10^{6}$ pairs of (*L*, θ). In all cases, the strongest peak occurs exactly at the location corresponding to the length of the short side of the flake (H/S=1 in all cases). The secondary peak appears at the location corresponding to the long dimension of the flake (H/S=r). This second peak progressively diminishes as *r* increases, and for r>5 it is practically undetectable. 100 bins were used for the generation of the histograms.

The frequency histograms for the intersection lengths can be computed from Equations (4)–(8). Figure 7 shows representative results for r=1,3,7, obtained by performing $10^{6}$ sets of calculations at each value of *r*, in which the distance *L* of the intersecting line and its orientation angle θ are taken as random variables uniformly distributed in the relevant intervals. The histograms generated from these calculations are identical with the results of the numerical sectioning experiments shown in Figure 3.

The explanation for the fact that the highest frequency occurs at H/S=1 can be found in the mechanism outlined in Figure 5 and presented in mathematical form in Equations (4)–(8). Consider a histogram with bin length (δ) and an intersection length $H_{n}$ corresponding to the *n*-th bin, such that $1+n\delta \;<\;H_{n}/S\;<\;1+\left(n+1\right)\delta$. From Equation (4), it then is

(9) $$1+n\delta \;<\;\frac{1}{\cos(\theta )}\;<\;1+\left(n+1\right)\delta$$

or equivalently

(10) $$\begin{array}{c} \theta_{n}\;=\;\mathrm{acos}\left(\frac{1}{1+n\delta }\right)\;<\;\theta \;<\;\mathrm{acos}\left(\frac{1}{1+(n+1)\delta }\right)\;=\;\theta_{n+1} \end{array}$$

The probability of having an intersection with length $H_{n}$, is proportional to the probability of finding an angle θ in the interval [$\theta_{n},\theta_{n+1}$]. This probability is

(11) $$\begin{array}{c} P\left[n\delta \;<\;\frac{H_{n}-S}{S}\right]∼\frac{\theta_{n+1}-\theta_{n}}{\pi }=\\ \frac{1}{\pi }\left(\mathrm{acos}\left(\frac{1}{1+(n+1)\delta }\right)-\mathrm{acos}\left(\frac{1}{1+n\delta }\right)\right) \end{array}$$

**Figure 8:**
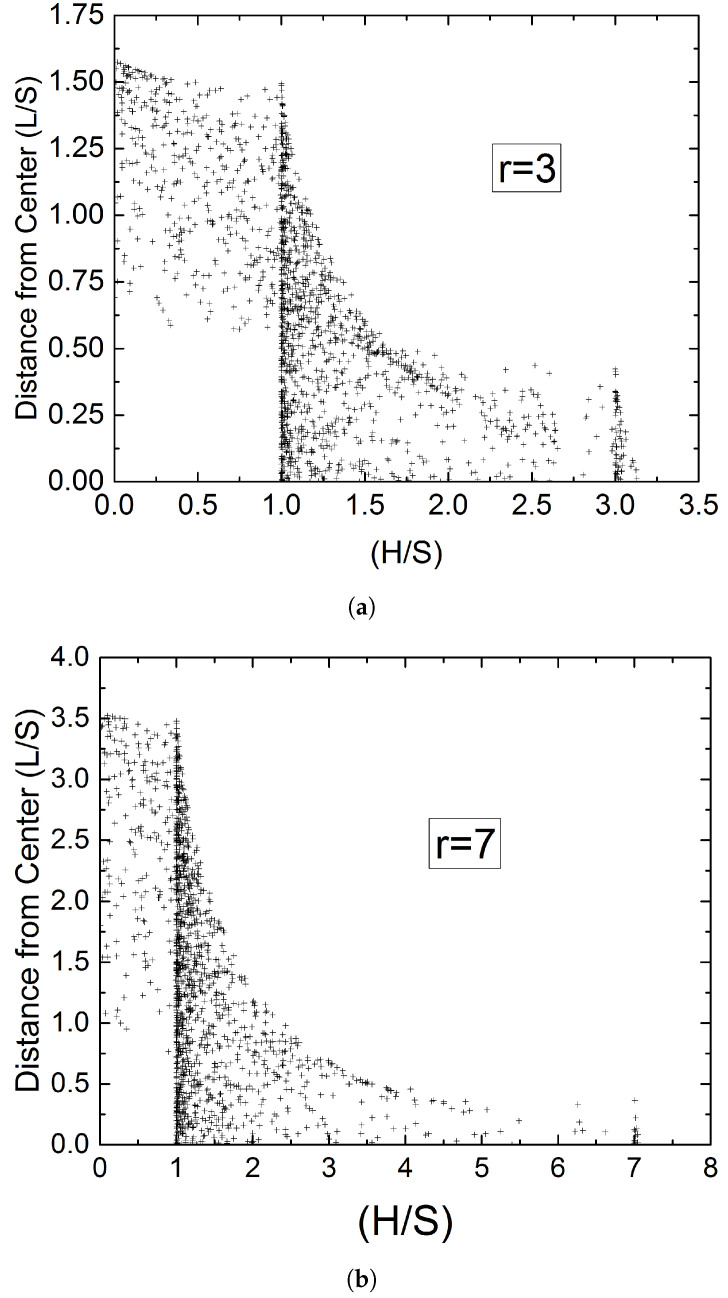
Correlation between length of intersections (*H*) and distance (*L*) of the intersecting line from the center of the flake—both normalized with *S*. Results obtained using the model of Equations (4)–(6) for a total of 2000 random combinations of (*L*) and (θ), which have resulted in 1642 (for r=3) (**a**) and 1428 (for r=7) (**b**) intersections. Each intersection corresponds to one point on the chart.

It can be easily verified that the probability expressed by Equation (11) is a decreasing function of *n*––the distance from the length H=S—and that it takes its maximum value at n=0, corresponding to H=S. Figure 8 shows the magnitude and frequency of intersection lengths *H* as function of the distance *L*. It is evident that planes intersecting a flake at small distances L<S/2 from its center give rise to long intersection lengths (lower right part of data points). On the other side, planes intersecting a flake at large distances from its center, give rise to shorter cuts, since they will intersect predominantly the two opposite short sides of the rectangle. Intersecting lines with H∼S correspond to planes intersecting at a very wide range of distances from the center of the rectangle; this multitude of intersections results in the histogram peak shown earlier in Figure 3 and Figure 7. Intersecting the two opposite short sides, which will result in long segments, corresponds to planes intersecting at small distances from the center; this is less probable for more slender flakes, resulting in the secondary peak diminishing with increasing *r* (compare concentration of points at H/S=3 and H/S=7 in Figure 8). This matter will be discussed further in following section.

### 4.2. Determination of the Flake Aspect Ratio from the Maximum Intersection Length

**Figure 9:**
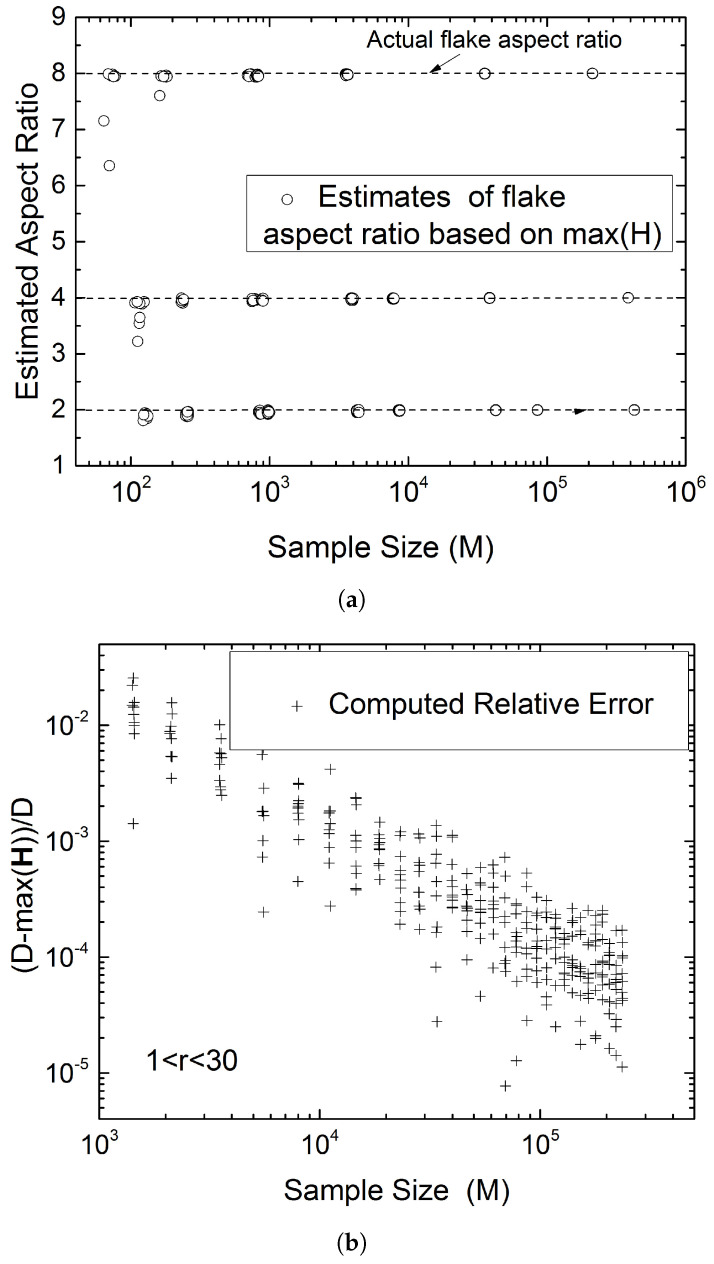
(**a**) Estimated flake aspect ratio r=W/S as function of sample size (*M*), defined as the number of flake intersections (lines) appearing in a cross-section. In each case, the short dimension of the flake (*S*) is determined from the first peak in the H-histogram (e.g., Figure 3), and the long dimension is determined from the diagonal D∼max(H) as $W=(\sqrt{D^{2}-S^{2}})$. (**b**) Summary of relative error as function of sample size (*M*) for a range of *r*.

The maximum possible intersection length is equal to the flake diagonal, or max(H)≤D, where **H** is the vector of intersection lengths. Given a sufficiently large sample size, it is conceivable that some intersection segments will satisfy H>(1−h)D, where *h* is a user-defined accuracy threshold. Calculating *W* from $D^{2}\;=\;W^{2}\;+\;S^{2}$ and using D∼max(H), can yield a quick conservative estimate of *W* and thus of *r*. While this approach appears, at first glance, to be highly empirical and uncertain, we show in this section that it produces estimates of the flake aspect ratio that are very close to the actual ones. Furthermore, by examining the related probabilities using Monte-Carlo simulations, based both, on numerical sectioning experiments and the model of Equations (4)–(8), we show that for a modest tolerance, e.g., h∼0.02, this will be achieved with sample sizes *M* no larger than $∼10^{3}$ and for certain flake aspect ratios with M as small as $∼10^{2}$. The following Figure 9 illustrates the performance of this method for various sample sizes and some indicative flake aspect ratios.

**Figure 10:**
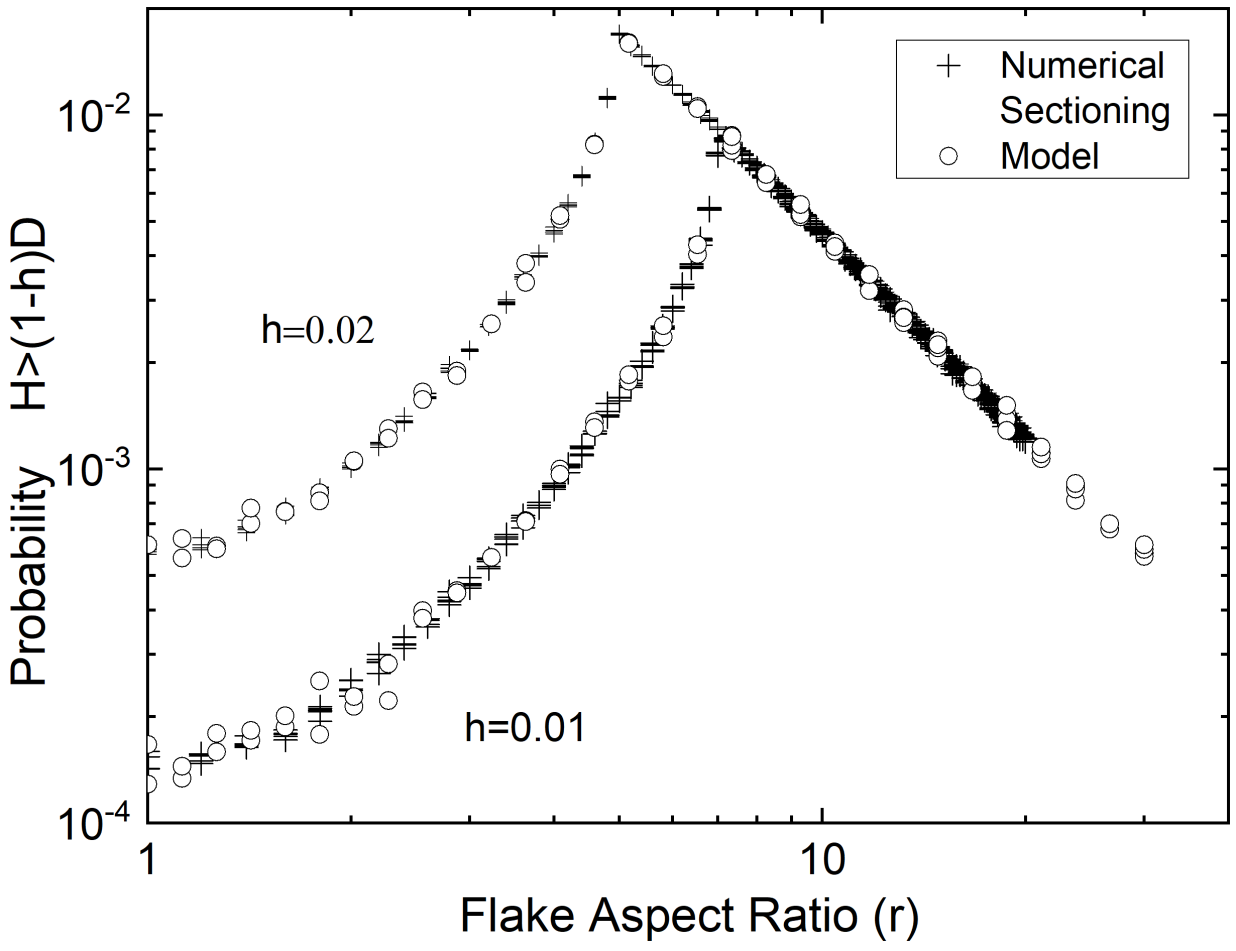
Comparison of the probability of having H>(1−h)D, as obtained from the model of Equations (4)–(8) (circles) and as obtained from numerical sectioning experiments in RVEs containing $∼10^{7}$ flakes (crosses).

The probability of achieving max(H)>(1−h)D was also computed by numerical sectioning experiments. These are compared to those obtained from the model of Equations (4)–(8) in Figure 10.

Further insight into this approach can be gained by examining Equations (4)–(8), and the distribution of *H* they generate, in a Monte-Carlo context. For long intersections to occur, it is clear that it must be L<S/2. From Equations (4)–(8) it is also clear that only intersecting the two opposite short sides of the rectangle (Equation (6), corresponding to $\theta_{2}\;<\;\theta \;<\;\theta_{3}$) or two adjacent sides (Equations (5) and (7), for $\theta_{1}\;<\;\theta \;<\;\theta_{2}$ and $\theta_{3}\;<\;\theta \;<\;\theta_{4}$ respectively) has any chance of generating long intersection lengths.

**Figure 11:**
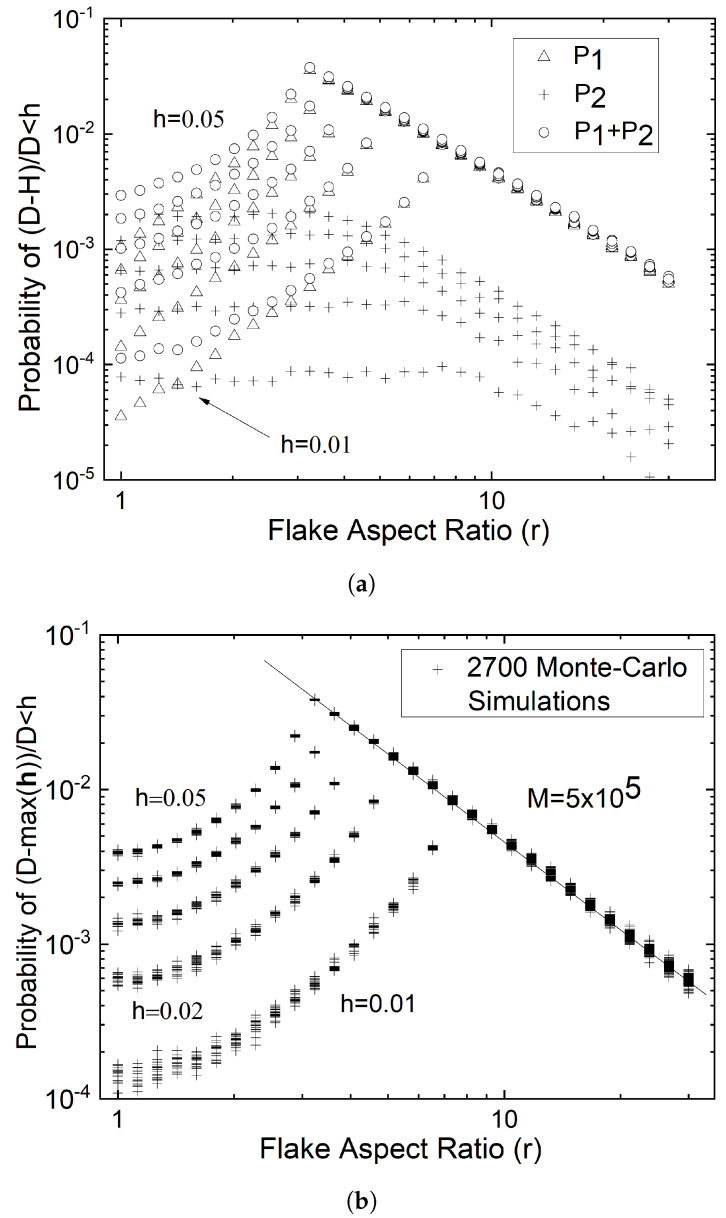
Monte-Carlo generated probabilities of obtaining H>(1−h)D. (**a**) One single scan for 1<r<30, with $M=2\times 10^{6}$ at each level of *h*, *h* = 0.01–0.05. (**b**) Results of 18 sets of simulations at each value of *h* (h=0.01n, n=1,2,...,5), scanning the range 1<r<30, for smaller sample size, showing the statistical variability of the related probabilities. The straight line passing through the points in the upper-right part of the graph has a slope of −1.90.

Figure 11 shows the probabilities of achieving H>(1−h)D for various values of *h*, as function of *r*, for each of the mechanisms outlined above. These probabilities are determined by Monte-Carlo simulations based on the model of Equations (4)–(8). For a number of random combinations of (*L*,θ), the total number of intersections achieved is *M*. The probability $P_{1}\left[H\;>\;\left(1-h\right)D\right]$ through the mechanism described by Equation (6) is $N_{1}/M$, where $N_{1}$ is the number of intersections caused by lines satisfying L<S/2 and $\theta_{2}\;<\;\theta \;<\;\theta_{3}$. The probability $P_{2}$ corresponding to the mechanism described by Equation (5) and Equation (7) (in this case, L<S/2 and $\theta_{1}\;<\;\theta \;<\;\theta_{2}$ or $\theta_{3}\;<\;\theta \;<\;\theta_{4}$) is $N_{2}/M$. The total probability of having H>(1−h)D is $P_{T}\left[H\;>\;\left(1-h\right)D\right]=\left(N_{1}+N_{2}\right)/M$. It is clear from Figure 9 that the mechanism described by Equation (6) is the most probable means of obtaining long intersection lengths. This probability is the highest for intermediate flake aspect ratios. The value of *r* corresponding to the maximum probability can be shown to depend on the desired tolerance and it is $r_{c}=\left(1-h\right)/(\sqrt{h(2-h)})$. For $r\;>\;r_{c}$ and also for $r\;<\;r_{c}$ the probability drops as a power function of *r*. For $r\;<\;r_{c}$, the probability approaches an asymptotic value at r=1; this value depends on *h*. These probabilities give some insight on the sample size M that might be required to achieve max(H)≥(1−h)D; assuming that $M\;∼P_{T}^{-1}$, *M* can range from $∼10^{2}$ to $∼10^{4}$, depending on the desired accuracy and the anticipated flake aspect ratio.

To summarize, up to this point we have shown that the length of the short side of the rectangle can be found from the histogram of the intersection lengths. When r<5, the length of the long side of the rectangle can also be inferred from the secondary peak in the histogram; since the strength of the secondary peak diminishes rapidly with increasing *r* another method is required to determine the flake aspect ratio from the same set of data when r>5. Such a method, that works well for larger sample sizes, is setting D∼max(H), where **H** is the vector of intersection lengths, and calculating *r* from $D/S=\sqrt{1+r^{2}}$. This will always underestimate the actual flake aspect ratio. An alternative method is presented in the following subsection.

### 4.3. Determination of Flake Aspect Ratio from the Average of the Intersection Lengths

**Figure 12:**
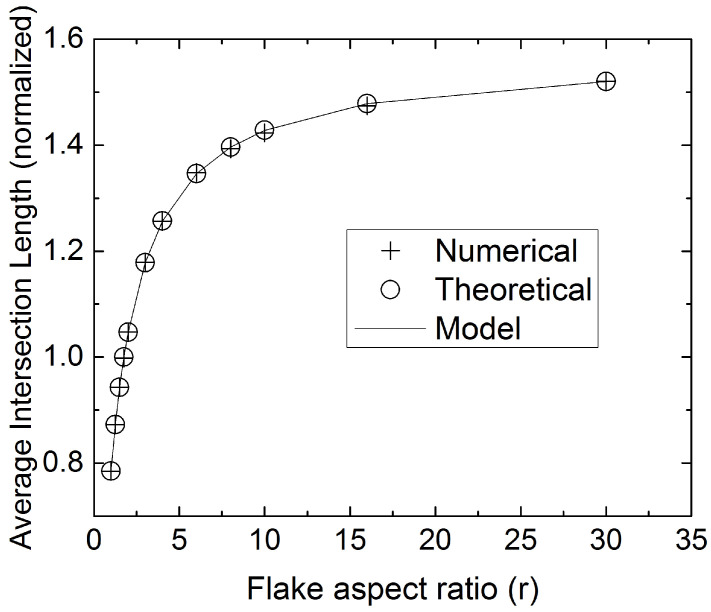
Computed (points) values of the average intersection length $H_{av}$, normalized by *S*, for various values of the flake aspect ratio, 1<r<30. The line shows the predictions of Equation (12). Both, predictions based on numerical sectioning of 3D RVEs (o) and model predictions based on Equations (4)–(8) (+) were obtained on samples having $M\;∼10^{6}$ flake intersections.

Once the distribution of intersection lengths is known, the average intersection length $H_{av}$ can be easily calculated, $H_{av}=\left(1/M\right)\sum H_{m}$, where *M* is the sample size. We will call $H_{av}$ “sample average”, to distinguish from the ensemble average 〈H〉 introduced below. $H_{av}$ can also be expressed in integral form from Equations (4)–(8), by averaging over all admissible (*L*) and (θ); since the limits of the required integrations (angles $\theta_{1}$–$\theta_{4}$) are functions of the random variable *L*, the result of this operation is cumbersome and can only be carried out numerically. In this subsection we adopt a direct Monte-Carlo approach in evaluating $H_{av}$ from Equations (4)–(8) as well as from numerical sectioning experiments. Through this it can be shown that $H_{av}$ is a function of *r* only. Figure 12 shows values of the $H_{av}$ obtained using numerical sectioning experiments as well as predictions based on Equations (4)–(8). The agreement between the two methods is excellent. Both sets of data are very well represented by the following model

(12) $$\frac{H_{av}}{S}=\frac{\pi r}{2(1+r)}\;$$

For very slender flakes, Equation (12) predicts that ($H_{av}/S$) approaches asymptotically the value of π/2.

Through Equation (12) the flake aspect ratio can be found from the average intersection length. In practice, given some uncertainty in the measured value of $H_{av}$ and the asymptotic behavior of $H_{av}$ at large *r*, the estimation accuracy of Equation (12) will be highest for small to moderate values of *r*, typically r<12; the accuracy of such estimates deteriorates rapidly for higher aspect ratios, as can be deduced from the value of the derivative $\partial r/\partial H_{av}$. It can be readily shown that $\Delta r/r=\left(1+r\right)\left(\Delta H_{av}/H_{av}\right)$, where Δr is the uncertainty in the estimation of *r*, given an uncertainty $\Delta H_{av}$ in the measurement of $H_{av}$.

**Figure 13:**
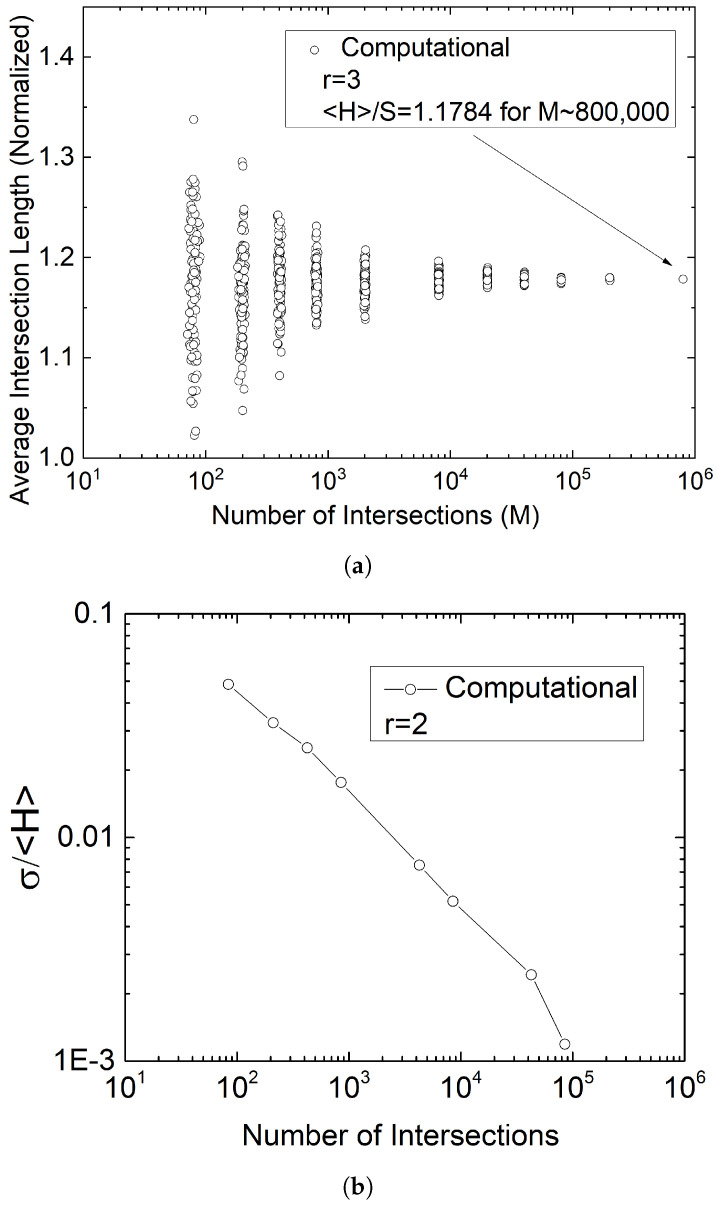
Effect of sample size M (horizontal axis) on (**a**) the predicted $H_{av}$ and (**b**) the normalized standard deviation for ensemble-averaging over 20 samples in each case (σ/〈H〉). Each point in (**a**) represents one sample.

Figure 12 and the resulting Equation (12) have been obtained using very large samples, $M\;∼10^{6}$. In practice, a sample obtained by sectioning a composite component may or may not contain this many flake images, depending on the dimensions of the sample and the number-density of the flakes in the part. Figure 13 shows the average intersection lengths $H_{av}$ computed from samples having from $M\;∼10^{2}$ to $M\;∼10^{6}$ flake segments. The ensemble average 〈H〉/S is defined as ($\langle H\rangle /S=\left(1/K\right)\sum H_{av,k}$ where *K* is the number of samples and $H_{av,k}$ is the average intersection length of the *k*-th sample, k=1,2,...,K. It is evident that while ensemble averages deviate very little as M varies over several orders of magnitude, their standard deviation is substantial at small sample sizes. It therefore appears that the method developed and proposed herein is best suited for large area samples, typically analyzed by large-area automated microscopy [29,30,31,32,33,34]. If single-frame images are to be analyzed, repeated sampling is necessary in order to obtain a reliable value for 〈H〉 and thus a reliable value of *r*. The ensemble-averaged values 〈H〉/S, for K=20 in each case and obtained from samples having on average 84, 211, 425, 852, 4270, 8545, 42,708, and 85,396 flake cross sections, are 1.0376, 1.0449, 1.0484, 1.0498, 1.0475, 1.0471, 1.0474, and 1.047, respectively. The value obtained from Equation (12) is 1.0470.

### 4.4. Comparison between Predictions and Results of Numerical Experiments

In the preceding subsections we presented methodologies that allow for calculation of the dimensions of rectangular flakes from knowledge of the statistics of the intersection lengths. The short dimension *S* of the flakes is obtained from the position of the major peak in the H frequency histogram (e.g., Figure 3 and Figure 7). The accuracy of this measurement depends entirely on the number of bins used in the histogram. There are three ways to evaluate the flake aspect ratio *r*. For r<5, it can be computed from the second peak in the intersection length histogram. For 1<r<12 it can also be found from the average intersection length (Equation (12)). A conservative estimate of *r* can also be obtained from the maximum intersection length (typically for $M\;>\;10^{3}$).

In the following, we carry out “blind” numerical experiments, in which the *H*-histogram and the sample $H_{av}$ and ensemble averages 〈H〉, as obtained from numerical sectioning, are given, and the flake dimensions *S* and W=Sr are computed and compared to the dimensions of the flakes in the systems from which the H-statistics were obtained. Based on the procedure outlined above, we obtain estimates of *r* ($r_{est}$ in Table 1). We will focus on the performance of the approach based on Equation (12) for 1<r<15.

**Figure 14:**
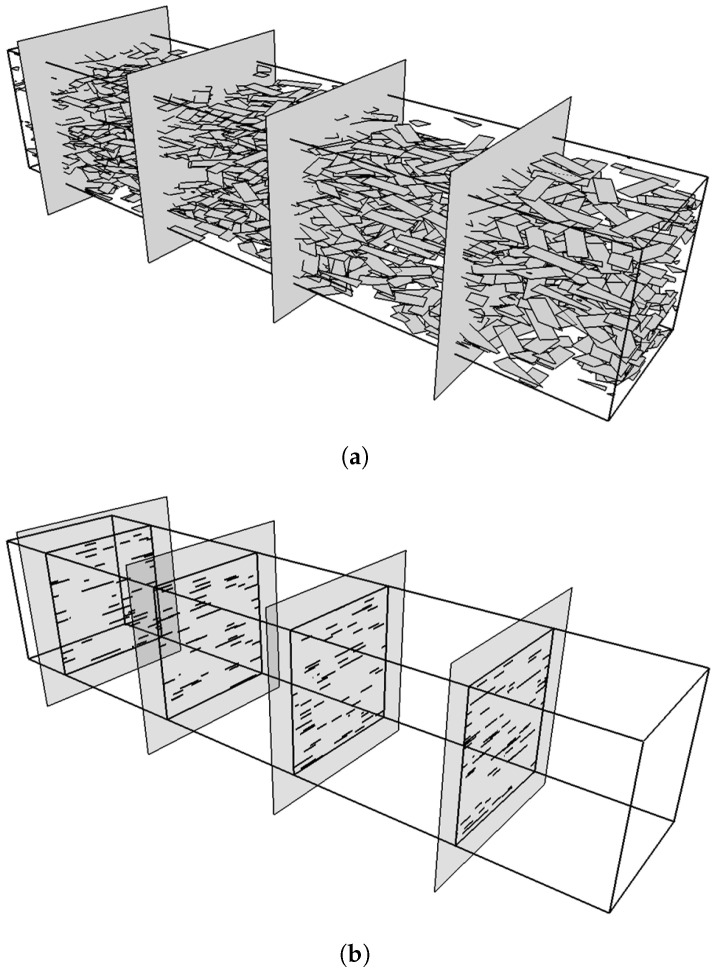
Schematic showing a numerical sectioning experiment. (**a**) 3D RVE containing flakes with their planes parallel to the *X*-*Z* plane and having random orientations in the *X*-*Y* plane. Also shown are the sectioning planes. (**b**) The sectioning planes showing only the flake intersections as lines of variable length. The number of the sections defines the ensemble size (here K=4). The number of intersections (lines) in each frame, defines the sample size (here M∼50 for clarity).

Numerical samples (3D RVEs) were generated by setting the value of flake number-density N/ΔV and flake width *S* and aspect ratio *r*. These are shown as $S_{actual}$ and $r_{actual}$ in Table 1. The thus generated geometries are then rendered blind, and numerical sectioning experiments are carried out to obtain the *H* statistics—histogram, $H_{av}$ for each sample and 〈H〉 for an ensemble of K=10 samples in each case. In Figure 14 the process is illustrated. It is obvious that for avoiding counting the same flake twice between cutting planes the distance between the cutting planes must be >2W.

Table 1 illustrates the performance of the method based on Equation (12) for flakes of various aspect ratios and for cross sections containing from $M\;∼10^{2}$ to $M\;∼10^{5}$ flake images. In each case 10 samples were obtained by sectioning at 10 different locations in each composite RVE and the 95% confidence intervals shown in Table 1 are computed as $\pm t(\sigma_{H}/\sqrt{K}$), with t=2.262 obtained from the Student’s distribution at K−1=9 degrees of freedom and $\sigma_{H}$ being the standard deviation of 〈H〉 computed from 10 samples.

**Figure 15:**
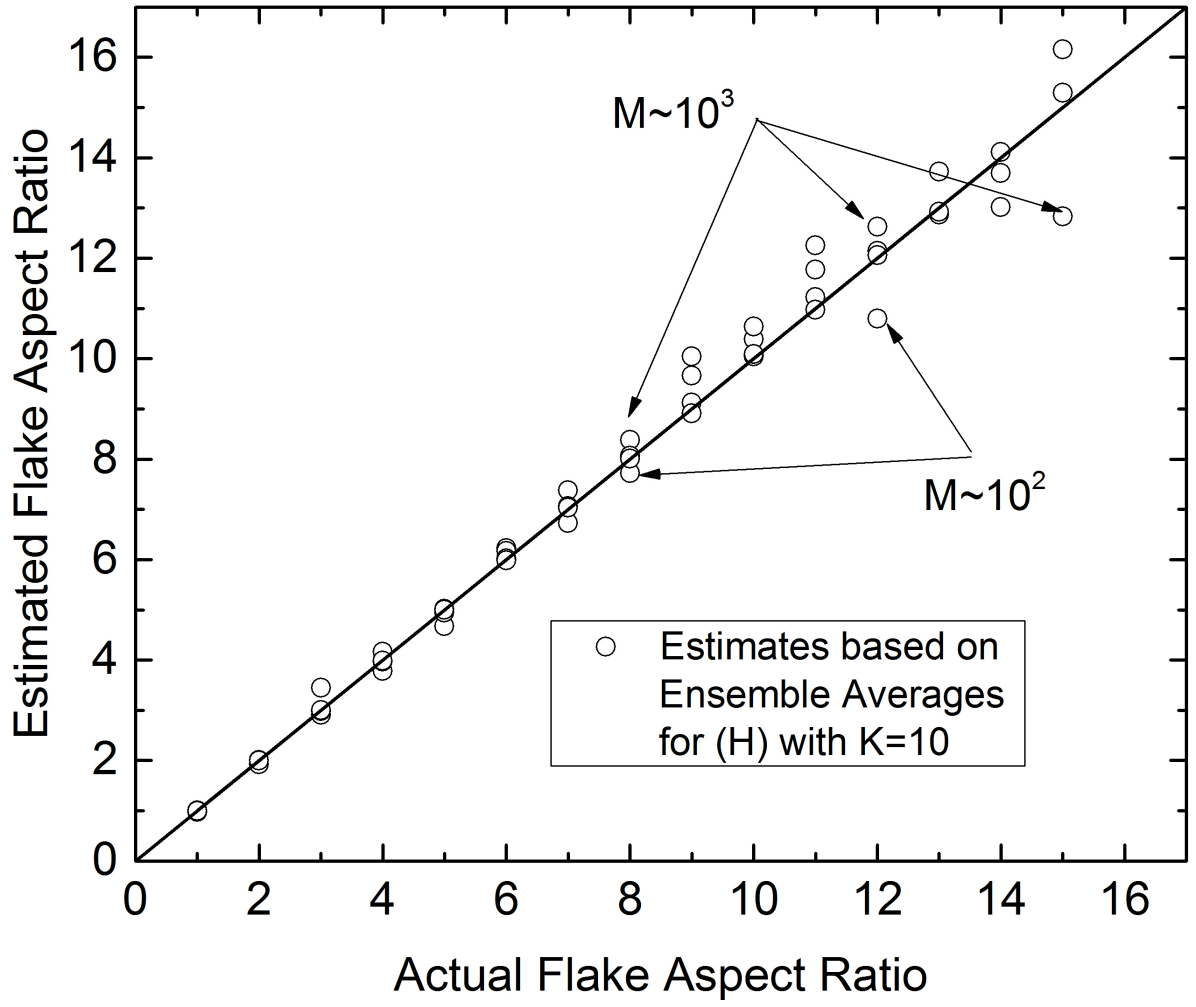
Parity graph between actual flake aspect ratio (horizontal axis) and estimated *r* from Equation (12) using ensemble-averaged values of $H_{av}$—obtained for K=10—and various sample sizes ($10^{2}\;<\;M\;<\;10^{5}$).

The accuracy of the predictions can be improved by increasing the ensemble size *K*, and for small sample sizes ($M\;∼10^{2}$) this is necessary at higher *r*. The following parity graph, Figure 15 illustrates further the performance of the method. It is seen that for a fairly modest ensemble size (K=10), the estimated flake aspect ratio is very close to the actual up to r=8, even with very small sample sizes ($M\;∼10^{2}$). It is clear that for $M\;>\;10^{3}$ the estimated flake aspect ratio agrees with the actual one for flakes having aspect ratios up to r=12.

## 5. Conclusions

We have presented a novel method for determining the size and aspect ratio *r* of rectangular flakes from the statistics of the length distributions seen in cross-sections of composite samples, such as those used in optical or electron microscopy, or obtained using tomographic imaging techniques.

Results of numerical sectioning experiments in realistic 3D RVEs containing up to $∼10^{6}$ flakes have shown that the primary peak of the length histogram corresponds to the short size of the rectangle while a secondary peak, evident at r<5, corresponds to the long dimension of the rectangle. These observations have been explained using Monte-Carlo simulations, as well as deterministic, geometry-based modeling and probability analysis. Therefore, for 1<r<5, the histogram of the intersection lengths allows us to derive the two principal dimensions of the flake. The accuracy of this measurement depends on the number of bins used in the histogram.

Since the strength of the secondary peak diminishes rapidly with increasing *r*, we develop two additional methods for the determination of *r*. The first method assumes D∼max(H), where H is the vector of intersection lengths, and calculates *r* from $D/S=\sqrt{1+r^{2}}$. This procedure is justified by computing the relevant probability fields using Monte-Carlo simulations, and estimates of the required sample sizes are derived, based on the required accuracy. While requiring large samples for flakes of large aspect ratio, this is the only method suitable for flakes with r>15. A second method for determining *r*, in the range 1<r<12, is based on a correlation we derive between the flake aspect ratio and the average length $H_{av}$ of the flake cross-sections; this method is accurate even for moderate sample sizes ($∼10^{3}$) for small *r*, requires larger sample sizes as *r* increases and becomes impractical for r>15.

## Figures and Tables

**Table 1:** Comparison between actual ($r_{actual}$) and back-calculated $r_{est}$ values of the flake aspect ratio, as obtained from measurement of $H_{av}$. The estimate of *S* is influenced solely by the number of bins used in the histogram of intersection lengths (100 bins were used in this example) and is 1 in all cases. ${}^{\left(1\right)}$ ensemble-average of K=10 samples; ${}^{\left(2\right)}$Δ〈H〉 is the 95% confidence interval according to Student’s distribution and is $\pm 2.262(\sigma_{H}/\sqrt{K}$); ${}^{\left(3\right)}$ estimates of *r* ($r_{est}$); ${}^{\left(4\right)}$Δr is the 95% confidence interval of the calculated values of *r* according to Student’s distribution.

$S_{\mathrm{actual}}$	$r_{\mathrm{actual}}$	*M*	$\langle H\rangle {\;}^{\left(1\right)}$	$\sigma_{H}$	$\Delta \langle H\rangle {\;}^{\left(2\right)}$	$r_{\mathrm{est}}{\;}^{\left(3\right)}$	$\Delta r{\;}^{\left(4\right)}$
1	1	130	0.7772	0.03429	0.025857	0.979	0.06451
		1250	0.7866	0.00765	0.005773	1.003	0.01475
		12,500	0.7851	0.00324	0.002443	0.999	0.00622
		128,000	0.7849	0.00071	0.000533	0.999	0.00135
1	2	125	1.0331	0.04439	0.033471	1.922	0.18200
		1230	1.0474	0.01480	0.011162	2.002	0.06405
		12,350	1.0481	0.00487	0.003671	2.006	0.02112
		127,000	1.0471	0.00106	0.000804	2.000	0.00461
1	3	120	1.2179	0.04845	0.036532	3.453	0.46120
		1180	1.1691	0.00857	0.00646	2.912	0.06293
		12,080	1.1763	0.00542	0.004087	2.983	0.04128
		121,000	1.1780	0.00222	0.001675	3.000	0.01707
1	4	115	1.2419	0.04515	0.034041	3.779	0.49493
		1130	1.2663	0.03251	0.024512	4.161	0.41566
		11,500	1.2548	0.00430	0.003242	3.973	0.05105
		116,000	1.2558	0.00241	0.001818	3.989	0.02882
1	5	110	1.2936	0.06103	0.046016	4.671	0.94229
		1120	1.3063	0.02227	0.016791	4.943	0.37765
		11,500	1.3097	0.00739	0.005576	5.021	0.12870
		116,000	1.3090	0.00249	0.001876	5.003	0.04305
1	6	105	1.3531	0.08089	0.060993	6.222	202.580
		1070	1.3516	0.01873	0.014119	6.171	0.46234
		11,000	1.3468	0.01087	0.008198	6.017	0.25705
		110,000	1.3459	0.00195	0.001473	5.989	0.04581
1	8	105	1.3905	0.10717	0.080805	7.723	391.494
		1060	1.4032	0.03815	0.028768	8.383	161.267
		10,550	1.3974	0.00511	0.003852	8.067	0.20164
		105,000	1.3963	0.00251	0.001892	8.012	0.09783
1	12	105	1.4375	0.09517	0.071758	10.80	636.127
		1070	1.4553	0.03430	0.025864	12.62	30.565
		11,000	1.4511	0.01171	0.008827	12.14	0.97103
		103,000	1.4503	0.00304	0.00229	12.06	0.24868
1	15	1030	1.4571	0.02617	0.019735	12.83	240.496
		10,600	1.4790	0.00762	0.005747	16.16	107.709
		101,000	1.4742	0.00548	0.004137	15.29	0.69910

## Data Availability

The raw/processed data required to reproduce these findings cannot be shared at this time as the data also forms part of an ongoing study.

## Abbreviations

The following abbreviations are used in this manuscript:

2D	two-dimensional
3D	three-dimensional
AFM	Atomic Force Microscopy
BIF	Barrier Improvement Factor
EMI	Electromagnetic Interference
RSA	Random Sequential Addition
RVE	Representative Volume Element
TEM	Transmission Electron Microscopes
